# KLK7 overexpression promotes an aggressive phenotype and facilitates peritoneal dissemination in colorectal cancer cells

**DOI:** 10.1002/2211-5463.70171

**Published:** 2025-12-03

**Authors:** Yosr Z. Haffani, Tobias Dreyer, Meriem Naim, Rea Lo Dico, Natalia A. Ignatenko, Viktor Magdolen, Dalila Darmoul

**Affiliations:** ^1^ Sorbonne Université CNRS UMR8263‐INSERM U1345, Institut de Biologie Paris Seine France; ^2^ Laboratory of Physiopathology, Alimentation & Biomolecules PAB, LR17ES03, Higher Institute of Biotechnology‐Sidi Thabet University of Mannouba Tunisia; ^3^ Clinical Research Unit, Department of Obstetrics and Gynecology Technische Universität München Germany; ^4^ National Cancer Institute Regina Elena (IRCCS) Rome Italy; ^5^ Department of Cellular and Molecular Medicine The University of Arizona Tucson AZ USA

**Keywords:** ascites, biomarker, colon cancer, integrin, Kallikrein‐related peptidase 7, metastasis, moesin

## Abstract

Colorectal cancer (CRC) incidence and mortality continue to rise globally and new prognostic biomarkers are required for the development of targeted therapies. Several studies have suggested that tissue kallikrein‐related peptidases (KLKs), including KLK7, contribute to tumorigenesis. We previously demonstrated KLK7's tumor‐promoting role both *in vitro* and *in vivo*, but its role in CRC metastasis remains unclear. Here, using the Cancer Genome Atlas (TCGA), we confirmed that KLK7 expression is upregulated in advanced stages of CRC and its association with shorter progression‐free survival (PFS) of patients. To further understand the role of KLK7 in CRC metastasis, we assessed its expression in ascites from CRC patients with peritoneal metastasis (PM), investigated cell behavior following KLK7 overexpression, and examined its role in metastasis using a mouse model. High KLK7 levels were found in malignant ascites, but not in benign ascites. In xenograft models, KLK7‐overexpressing cells increased PM and exhibited higher Peritoneal Cancer Index (PCI) scores compared to controls. *In vitro*, KLK7 overexpression in HT29‐D4 human colon cancer cells significantly enhanced cell proliferation, colony formation, migration, spheroid formation, and adhesion to extracellular matrix proteins. Additionally, KLK7 overexpression altered cell morphology, upregulated moesin (MSN) and integrin subunits, suggesting cytoskeletal remodeling and matrix interactions. Taken together, these findings suggest that KLK7 is a driver of CRC progression and could serve as a potential prognostic marker for aggressive forms of CRC.

## Graphical abstract

KLK7, a tissue kallikrein‐related peptidase, is elevated in advanced colorectal cancer and associated with shorter survival. High KLK7 levels in ascites correlate with peritoneal metastasis. In mice, KLK7 overexpression increases metastasis. *In vitro*, KLK7 enhances cancer cell proliferation, migration, adhesion, and spheroid formation, driving cytoskeletal changes. KLK7 thus contributes to colorectal cancer progression and may serve as a prognostic marker.

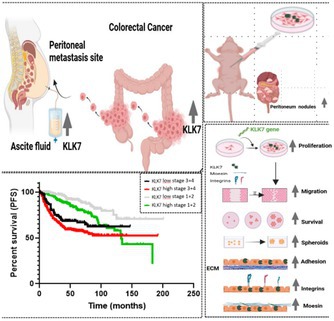

**Abbreviations**
BSA: bovine serum albumin
CMS4: consensus molecular subtype 4
COAD: colon adenocarcinoma
COX: cyclooxygenase
CPM: colorectal peritoneal metastasis
CRC: colorectal cancer
CYR61: cysteine‐rich angiogenic inducer 61
DAPI: 4',6‐diamidino‐2‐phenylindole
DFS: disease‐free survival
DMEM: Dulbecco's modified eagle medium
ECM: extracellular matrix
ELISA: enzyme‐linked immunosorbent assay
EpCAM: epithelial cell adhesion molecule
ERM: ezrin‐radixin‐moesin
FCS: fetal calf serum
GAPDH: glyceraldehyde‐3‐phosphate dehydrogenase
GEO: gene expression omnibus
IGFBP6: insulin‐like growth factor binding protein 6
KLK: kallikrein‐related peptidase
KM Plotter: the kaplan meier plotter
KRT19: keratin 19
MMP: matrix metalloproteinase
MSN: moesin
NML: nonmalignant liver
OS: overall survival
PCI: peritoneal cancer index
PM: peritoneal metastases
RFS: recurrence‐free survival
RT‐PCR: Reverse Transcription Polymerase Chain Reaction
SD: standard deviation
TCGA: The Cancer Genome Atlas
UICC: International Union Against Cancer

Colorectal cancer (CRC) incidence and mortality are globally rising. Despite advances in diagnostics and treatment, survival rates for metastatic CRC remain poor due to high recurrence rates and the cancer's strong propensity to spread [1]. In addition to liver and lung metastases, peritoneal metastases (PM) represent one of the most challenging manifestations of CRC, characterized by cancer cell dissemination to the peritoneal lining and often accompanied by the accumulation of malignant ascites [2]. PM is typically diagnosed at advanced stages, leading to serious complications, limited treatment options, and poor clinical outcomes. Current biomarkers do not reliably predict recurrence, metastasis, chemotherapy resistance, or reduced survival, which hampers effective therapeutic decision‐making. Thus, identifying new prognostic biomarkers is essential for developing targeted therapies and improving patient outcomes.

There is growing evidence that proteases contribute significantly to cancer progression through multiple mechanisms, including the cleavage of cell adhesion molecules, growth factors, transmembrane receptors, and cytokines [3]. Notably, a subset of serine proteases can also function as signaling molecules by interacting with protease‐activated receptors (PARs) on the cell surface [4, 5, 6]. Among these proteases, the human kallikrein‐related peptidase (KLK) family comprises 15 serine endopeptidases (KLK1–15), expressed in a wide range of tissues and involved in numerous physiological processes such as skin desquamation, semen liquefaction, and neural plasticity [7]. Importantly, KLKs have been implicated in a variety of pathological conditions, including inflammatory diseases and cancer [4, 8, 9]. Dysregulation of KLKs has been reported in several malignancies, underscoring their role as key mediators of tumorigenesis [4, 8]. KLKs contribute to cancer progression by promoting tumor growth, migration, invasion, angiogenesis, and chemoresistance [10, 11]. These effects are mediated through remodeling of the extracellular matrix (ECM) and modulation of the tumor microenvironment, as well as through proteinase‐activated receptors (PAR)‐mediated cell signaling [5, 6].

KLK7 expression has been detected in various cancers, particularly in CRC. The Cancer Genome Atlas (TCGA) RNA sequencing data analysis across 15 different cancer types identified KLK6, KLK7, KLK8, and KLK10 as promising diagnostic biomarkers for colon adenocarcinoma [12]. A recent meta‐analysis by Peng and colleagues [13], confirmed that elevated KLK6, KLK7, and KLK10 levels are significantly associated with poor overall survival (OS) and shorter disease‐free survival (DFS) in CRC patients [13].

KLK7, a protein predominantly expressed in the skin and primarily recognized for its role in desquamation [9], is often dysregulated in various cancers, including CRC [4, 7, 8]. Our previous research demonstrated that KLK7 is markedly overexpressed in CRC tissues compared to normal colonic mucosa [14]. Elevated KLK7 expression has been associated with poor clinical outcomes in the colon [15, 16], ovarian [17], and pancreatic cancers [18, 19], whereas in breast cancer, it has been associated with a more favorable prognosis [20].

As a result, KLK7 has emerged as a potential prognostic biomarker, with elevated expression in most tumor entities correlating with more aggressive tumor behavior. One proposed mechanism underlying its oncogenic role involves its enzymatic degradation of extracellular matrix (ECM) components, thereby facilitating cellular invasion and metastatic spread. Nevertheless, its exact role in driving metastatic progression, especially in CRC, remains poorly defined.

A prominent indicator of metastatic colorectal cancer (CRC) spreading to the peritoneum is the development of ascites, an abnormal accumulation of fluid in the abdominal cavity. This occurs as tumors disrupt the peritoneal environment by increasing blood vessel permeability, obstructing lymphatic drainage, and altering nearby tissues. Ascites is frequently observed in cases of peritoneal carcinomatosis, leads to a significant decline in quality of life, and is strongly correlated with decreased overall survival [21].

Recent research suggests that these pathological changes in the peritoneal environment may be driven, at least in part, by molecular regulators of metastasis such as moesin (MSN). MSN, a member of the ERM (ezrin‐radixin‐moesin) protein family, has been identified as a key player in peritoneal metastasis, a critical event in the progression of gastrointestinal and ovarian cancers [22]. Elevated MSN expression has been associated with poor patient outcomes across various cancer types and is thought to promote cancer progression through its roles in cytoskeletal remodeling, cell shape regulation, and enhanced migratory capacity [23].

This study is the first to identify elevated levels of KLK7 protein in the ascitic fluid of colorectal cancer (CRC) patients with peritoneal metastases, distinguishing it from benign ascites. Overexpression of KLK7 in xenograft models was shown to promote peritoneal metastasis. *In vitro* experiments demonstrated that KLK7‐overexpressing HT29‐D4 colon cancer cells exhibited increased proliferation, colony formation, migration, multicellular aggregate formation, and adhesion to extracellular matrix components. These functional changes were accompanied by morphological alterations, increased MSN expression, and upregulation of specific integrin subunits. These findings suggest that KLK7 plays a significant role in enhancing metastatic traits in colorectal cancer, highlighting its potential as a target for therapeutic intervention and as a biomarker for metastatic progression.

## Materials and methods

### Tissue immunohistochemistry

Immunohistochemistry was performed on archival formalin‐fixed paraffin‐embedded tissue samples with colonic adenocarcinomas (Pathology Department of the Bichat‐Claude Bernard Hospital, Paris) and has been utilized here to analyze KLK7 expression in relation to cancer staging. The samples were used in accordance with the requirements of the Human Research Committee of the Bichat‐Claude Bernard Hospital and according to the Declaration of Helsinki, which is adopted by French Bioethical Law [24]. Tumors were staged according to the 7th International Union Against Cancer (UICC) Edition. Immunostaining was performed using a Menarini Bond Max automat (Rungis, France) as described in [14, 25]. KLK7 staining was assessed by two independent observers and the percentage of immunostained epithelial cancer cells was evaluated employing a semi‐quantitative method. Staining intensity was scored as: 0, negative; 1, weak; 2, moderate; 3, strong; 4, intense. Results of scores were obtained by multiplying the percentage of positive cells by the intensity.

### Survival analysis of samples with overexpressed KLK 7

To explore the role of kallikrein‐related peptidase 7 (KLK7) in colon cancer, we conducted an *in silico* analysis using publicly available datasets from The Cancer Genome Atlas (TCGA). The data retrieval and analysis were conducted through the KM Plotter interface.

The study utilized normalized KLK7 mRNA expression data from TCGA colon adenocarcinoma (COAD) samples to analyze its expression across the different clinical stages. Patients were stratified into early‐stage (stage 1 + 2) and advanced‐stage (stage 3 + 4) groups, and the Mann–Whitney *U*‐test was employed to compare KLK7 expression levels between these groups, aiming to identify differences associated with tumor progression.

To assess the prognostic significance of KLK7, patients were divided into high‐ and low‐expression groups based on the median KLK7 expression value. Kaplan–Meier (KM) survival curves were generated to analyze progression‐free survival (PFS), and the log‐rank test was used to determine the statistical significance of survival differences.

### Cell culture

The HT29‐D4 cell line, a clone derived from the human colon cancer cell line HT29, was a gift from Dr. J. Marvaldi (Université d'Aix‐Marseille, France). The human HT29 cell line was previously shown not to express KLK7 [14]. This absence of endogenous KLK7 expression makes the clone HT29‐D4 an ideal model for overexpression studies, as it allows for the evaluation of KLK7‐specific effects without background interference. Cells were verified for their authenticity and were routinely checked for free mycoplasma contamination (Biovalley, Nanterre, France). Cells were maintained at 37 °C in a humidified atmosphere of 5% CO_2_/air in DMEM containing 4.5 g·L^−1^ glucose, supplemented with 10% FCS (Life Technologies, Cergy‐Pontoise, France).

### Cell transfection and selection

The empty vector pRc/RSV (Invitrogen, Dreieich, Germany) and plasmids harboring either the coding region of KLK7 (pRc/RSV‐KLK7) or the coding sequence with a serine to alanine mutation in a catalytic triad (pRc/RSV KLK7‐S195A) were transfected into HT29‐D4 using the lipofectamine 3000 (Invitrogen, Carlsbad, CA, USA) according to the manufacturer's instructions. This vector was chosen because it contains the Rous sarcoma virus (RSV) promoter, which enables robust and constitutive expression in mammalian cells, and includes a neomycin resistance gene for effective selection using G418. Forty‐eight hours after transfection, a selective medium containing G418 (2 mg·mL^−1^) was added to the transfected cell cultures. Two weeks after transfection, G418‐resistant colonies were selected and expanded. The pool of neomycin‐resistant KLK7‐transfected HT29‐D4 cells was denoted as HT29‐D4‐KLK7. HT29‐D4 cells, stably transfected with the vector only, were designated as HT29‐D4‐Vector cells.

### Quantitative reverse transcription PCR

KLK7 mRNA levels in the transfected cell lines were determined by quantitative real‐time PCR. PCR reactions were performed in duplicate using the Power SYBR^®^ Green PCR Master Mix kit (Roche) following the manufacturer's instructions, using the following primers: 5′CCCAGTGCTCTGAATGTCAA3′ (forward) and 5′AGTGGGAATCTCGTTCATCC3′ (reverse) for KLK7; 5′TGGGTGTGAACCATGAGAAGTATG3′ (forward) and 5′GGTGCAGGAGGCATTGCT3′ (reverse) for GADPH, a housekeeping gene used as an internal standard. One microgram of total RNA was reverse transcribed using the ThermoScript RT‐PCR kit (Invitrogen, Carlsbad, CA, USA) according to the manufacturer's instructions. The final 10 μL reaction volume includes 5 μL of 2 × Power SYBR^®^ Green PCR Master Mix, 50 nm of each primer and 2 μL cDNA. Both the *KLK7* target gene and the *GAPDH* reference gene sequences were amplified in separate duplicate reactions for each sample and the average *C*_*T*_ value was calculated. The conditions for PCR were as follows: 10 min at 95 °C, then 40 cycles of amplification at 95 °C for 15 s and 60 s at 60 °C. All samples were normalized to the expression of the housekeeping GAPDH gene. Relative quantification of the target gene expression was done using the comparative cycle threshold (*C*_*t*_) method according to the manufacturer's instructions. *C*_*t*_ was normalized to GAPDH (Δ*C*_*t*_ = *C*_*t*_ sample − *C*_*t*_ GAPDH).

### Animals

Female BALB/c nude mice (five‐week‐old) were obtained from Charles River Laboratories (Saint Germain sur Arbresle, France), housed in a specific pathogen‐free compliant animal facility and were first acclimated for 2 weeks.

All of the experimental protocols met the standards required by the European Community guidelines for the care and use of laboratory animals. The laboratory number for the national accreditation for animal experimentation is C75‐10‐03 (November 27, 2012). The project was submitted to the French Ethic Committee and obtained the authorization APAFIS#24071‐2 020 020 611 035 354 v4.

### *In vivo* mouse model for PMs

HT29‐D4‐KLK7 and HT29‐D4‐D4‐vector cells (1 × 10^6^ cells) were injected intraperitoneally (*i.p*.) into BALB/c nude mice (*n* = 10 mice/group) as a model for peritoneal metastasis [26]. BALB/c nude mice offer a cost‐effective and immunodeficient environment that supports reliable engraftment of HT29 human colorectal cancer cells, making them well suited for studying preclinical research. Mice were monitored and weighed twice a week. Forty‐two days after injection, the mice were sacrificed by cervical dislocation under general anesthesia (2% isoflurane), and then checked for evidence of peritoneal metastasis. The peritoneum was photographed and the number of macroscopic nodules quantified in the peritoneal cavity applying a Peritoneal Cancer Index (PCI) [27]. In brief, the abdominal cavity was opened and virtually divided into 13 distinct regions (Fig. S1). A score from 0 to 3 was allocated for every region, and the sum of the 13 scores gave the total PCI for each mouse. A score = 0 corresponds to no macroscopic tumors; a score = 1 corresponds to 1–2 nodules in the region, with the size of nodules < 2 mm; a score = 2 corresponds to 1–2 nodules, and with the nodules' size between 2 to 4 mm; a score = 3 corresponds to more than 10 nodules with the nodules' size > 4 mm. The modified PCI as used in this murine model was calculated by adding the scores of all regions. A PCI below 10 represents a limited carcinomatosis, a PCI between 11 and 20 an intermediate carcinomatosis and a PCI above 20 an advanced carcinomatosis [28].

### Immunofluorescence staining

Immunofluorescence detection was performed with cells grown on glass coverslips (IBD). Cells were washed three times in PBS, fixed in 2% paraformaldehyde, washed three times in PBS, then incubated with PBS containing 2% BSA for 15 min and permeabilized with 0.1% Triton X. For the detection of F‐actin, fixed, permeabilized cells were incubated with rhodamin‐phalloidin (5 units·mL^−1^), (Life Technologies, Cergy‐Pontoise, France) for 30 min at room temperature. Finally, the cells were mounted in Vectashield medium containing DAPI dye (Vector, Peterborough, UK) and examined using a fluorescence microscope and confocal analysis (LSM 510 Zeiss, Jena, Germany) (magnification 630×).

### Peritoneal fluid

Ascitic fluids (*n* = 13) were collected from adult patients with peritoneal metastasis from colorectal cancer (CPM), or from control ascites from nonmalignant liver diseases (alcoholic cirrhosis) (NML, *n* = 5). The patients consented to the sample collection, and all individual patient‐related reports and data were anonymized before processing for the samples biobanking. Ascitic fluid extraction is a part of the routine management of patients, and informed consent for ascites analysis was obtained from each patient prior to surgery.

The peritoneal fluids obtained were submitted to a short spin at 2000 rpm for 10 min to separate the cells from ascites supernatants. The supernatants were aliquoted and stored for further use at −80 °C until analysis.

### Enzyme‐linked immunosorbent assay (ELISA)

KLK7 antigen was assessed using a non‐competitive immunoassay as previously described [25]. Colon cancer cells were seeded at 40 000 cells/well in 12‐well plates. At confluence, the conditioned medium was collected for antigen determination of KLK7. Then, the cells were detached and counted. Protein values represent the mean concentration of KLK7 expressed by 10^6^ cells from three independent experiments performed in triplicate. For ascitic fluids, protein values represent the mean concentration of KLK7 expressed per liter. Ascitic fluid extraction is a part of the routine management of patients, and informed consent, for ascites analysis, was obtained from each patient prior to surgery. All information related to the used surgical material was recorded in such a manner that subjects cannot be identified, directly or through identifiers linked to the subjects. The performed study does not involve interaction or intervention with a living individual or group of individuals; the study does not involve access to identifiable private information. The provider of the specimens removed the code before sending the specimens for this study and the investigators only received specimens that are fully de‐identified. Therefore, the Human Subjects Research reported in this manuscript is not considered clinical research and does not require Institutional Review Board approval.

### Cell proliferation assays

Cell proliferation was monitored by using the xCELLigence real‐time cell analysis (RTCA) (Agilent Technologies, France). xCELLigence cell index (CI) impedance measurements were performed according to the supplier's instructions. Cells were resuspended in the medium and subsequently adjusted to 20 000 cells·mL^−1^. After seeding, 100 μL of the cell suspensions into the wells of the E‐plate 96, the cell proliferation index was assessed every 15 min for a period of up to 96 h by the xCELLigence system.

### Colonogenic assay

The colony formation assay, also known as the colonogenic assay, is a well‐established method used to assess the ability of a single cell to grow into a colony. Briefly, cells were placed in the plates (six‐well) at a low density (1000 cells/well) and allowed to generate single colonies for 14 days. The colonies were washed twice in PBS, then stained with 0.5% (V/V) crystal violet‐20% methanol, imaged using an Azure™ Imaging System, and quantified with the ImageJ software (GE Healthcare, Piscataway, NJ). At least three independent experiments were performed in duplicate.

### Spheroid formation

Spheroids were generated by growing cells at 1000 cells·mL^−1^ in ultra‐low attachment 6‐well plates (ThermoFisher™ Scientific, Fontenay‐sous‐Bois, France) in SM (sphere‐formation medium consisting of DMEM GlutaMAX (0.5×; Gibco^®^), supplemented with B27, 20 ng·mL^−1^ epidermal growth factor, 20 ng·mL^−1^ basic fibroblast growth factor, and 2 mm L‐glutamine (ThermoFisher™ Scientific, Fontenay‐sous‐Bois, France) on ultra‐low attachment plates (Merck Millipore S.A.S., R&D Hub Molsheim, Molsheim, France). Conditioned medium (CM) was changed every 3–4 days. The spheres were typically counted after 14 days. Spheres were imaged using the EVOS auto‐imaging system (ThermoFisher™ Scientific).

### Cell migration assay

Migration assays were performed using culture‐insert‐μDish (ibid, Martinsried, Germany) of two chambers (growth area per well 0.22 cm^2^) separated by a wall (width: 500 μm). Briefly, a culture insert was placed into the 6‐well plate. The cells (7.5 × 10^5^ cells·mL^−1^; 80 μL) were seeded into each well in 10% (v/v) fetal calf serum‐containing medium. After attachment of the cells overnight at 37 °C in culture, inserts were gently removed to form the cell‐free gap. Then, cells were preincubated in the presence of 25 μg mitomycin for 30 min and subsequently cultured in serum‐free medium. An inverted phase‐contrast microscope was used to capture the images at 0, 24, and 48 h using the EVOS auto‐imaging system microscope. Five randomly chosen fields from 48 h migrated samples were used to calculate the percentage of wound closure in the cell‐free gap using the ImageJ software. Wound healing assays were performed in three independent experiments (*n* = 6).

### Cell adhesion assay

Adhesion assays were developed using a colorimetric ECM Cell Adhesion Array Kit (Merck Millipore S.A.S.) in triplicates for each cell culture condition, following instructions by the manufacturer (Merck Millipore S.A.S.). Briefly, HT29‐D4‐KLK7 and HT29‐D4‐Vector cells were seeded at 10^5^ cells/well in 96‐well tissue culture plates coated with type I collagen (Col‐I), type II collagen (Col‐II), type IV collagen (Col‐IV), fibronectin (FN), laminin (LN), tenascin (TN), or BSA (B) as a negative control. Cells were incubated for 2 h at 37 °C. Thereafter, adherent cells were washed 3 times with PBS, and the attached cells were fixed, stained with crystal violet, and solubilized. Their optical densities were measured at 570 nm (OD_570_). The assay was performed in triplicates. Means of three independent experiments are presented.

### Identification of cell surface integrins

The Integrin‐Mediated Cell Adhesion Array EMC535 (Merck Millipore S.A.S.), an assay which can substitute FACS analysis, was used for the characterization of alpha and beta integrin surface expression on HT29‐D4‐KLK7 and HT29‐D4‐Vector cell surfaces. Briefly, the HT29‐D4‐KLK7 and HT29‐D4‐Vector cells were gently detached with accutase, washed, and seeded in triplicates for each cell culture condition at 10^5^ cells/well in serum‐free medium in 96‐well tissue culture plates. Cells were then incubated for 2 h at 37 °C. Non‐bound cells were washed 3 times with PBS, and the attached cells were fixed, stained with crystal violet, and solubilized following instructions by the manufacturer (Merck Millipore S.A.S.); and the optical densities were read at 540 nm (OD_540_). The assay was performed in triplicates. Means of three independent experiments are presented.

### Western blot analysis

Cells were lysed either with RIPA buffer or with Mem‐PER Plus Membrane Protein Extraction Kit for membrane integrin expression, supplemented with EDTA‐free Protease Inhibitor Cocktail (Roche SAS, Boulogne‐Billancourt, France ). Total protein concentration was determined using the BCA Kit.

Equal amounts of solubilized membrane proteins (30 μg) were separated by SDS/PAGE and transferred onto a nitrocellulose membrane. Membranes were incubated overnight at 4 °C in blocking buffer (20 mm Tris, 50 mm NaCl, and 0.1% (v/v) Tween 20 (TBST) containing 5% (w/v) low‐fat milk or 5% BSA/TBST) according to the manufacturer. Before antibody hybridization, the membrane was cut according to the size of the protein ladder to ensure precise size determination. After blocking, the membrane was incubated with the primary antibody against the target protein (dilution in 5% BSA/TBST or 5% low‐fat milk) and then probed overnight at 4°C with the following antibodies: moesin (#26053‐1AP; Proteintech, Martinsried, Germany; dilution: 1 : 5000) and integrin αv (#4711), integrin β3 (D7X3P) XP (#13166) and integrin β5 (D24A5) (#3629) in the integrin sampler kit (Cell Signaling Technology, Inc., Massachusetts, USA) (dilution: 1 : 1000). The following day, the membrane was washed three times with TBST and incubated with the secondary antibody conjugated with horseradish peroxidase (HRP) (1 : 2000 dilution) for 1 h at room temperature. Membranes were stripped in stripping buffer (Invitrogen), reprobed with a monoclonal beta‐actin antibody (#A5441; dilution: 1 : 5000) and then incubated with mouse HRP‐linked secondary antibody (dilution: 1 : 5000; Merck, Darmstadt, Germany) as loading controls. Proteins were revealed by applying the Signal^®^ Chemiluminescent Substrate (Invitrogen) on a Chemi‐Doc‐MP imaging system (Bio‐Rad, Marnes‐la‐Coquette, France).

### *In silico* dataset

The mRNA expression data of CRC patients were derived from the KM Plotter website on 09.07.2025 https://kmplot.com/ [29] Acquisition was performed for the probe id 239381_at with stratification to RFS and stages 1 + 2 vs 3 + 4. All available GSE datasets were used for the final analysis.

### Statistical analysis

Analyses were performed using Prism 6 (GraphPad Software Inc., La Jolla, CA, USA). Experiments were performed at least in triplicate and results are expressed as mean ± standard deviation (SD). For comparisons between two independent groups, the Mann–Whitney *U*‐test (a nonparametric statistical test) was used to evaluate differences. For extracellular matrix (ECM) cell adhesion assays and integrin expression array data, normality was assessed using the Shapiro–Wilk test, and statistical comparisons were performed using an unpaired *t*‐test with Welch's correction. A *P*‐value < 0.05 was considered statistically significant (NS > 0.5, *P < 0.05, **P < 0.01, ***P < 0.001, ****P < 0.0001).

## Results

### Expression of KLK7 is associated with higher tumor stage in colorectal cancer

**Fig. 1:**
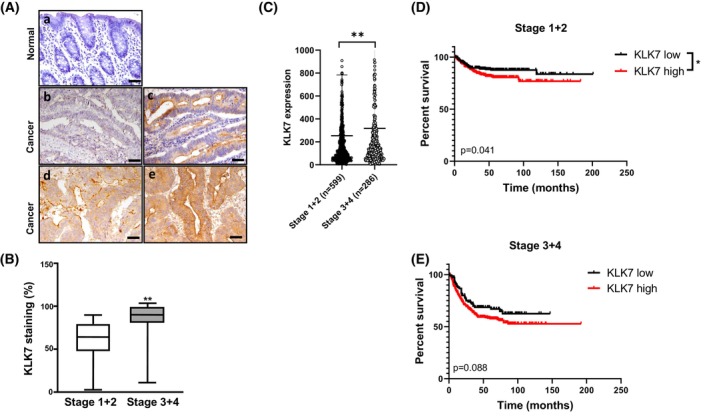
Correlation of KLK7 expression levels with the pathological stage of CRC patients. (A) Representative immunohistochemical (IHC) staining of KLK7 in human CRC tissues. Negative or discrete KLK7 staining was observed in non‐CRC tumor‐adjacent tissues (a). IHC staining revealed visible expression of KLK7 in CRC tumor tissues at stages 1 and 2 (b and c), strong staining was present at stages 3 and 4 (d and e). Bars: 50 μm. (B) Whisker plot representing the percentage of KLK7 staining in relation to the tumor stage. Tumors were staged according to the TNM classification. Staging was done according to the pathological records. Data represent mean ± SD. A total of 21 cases were grade 1–2 and 16 cases were grade 3‐4. Mann–Whitney *U*‐test was employed to compare KLK7 expression levels between stage 1 + 2 and stage 3 + 4 groups. **P < 0.01. (C) KLK7 mRNA expression increases with the progression of colon cancer stages. Box plot illustrating elevated KLK7 mRNA expression in colon cancer tissues at stages 3 and 4 compared to stages 1 and 2. The expression levels of KLK7 are represented as FPKM values derived from an *in silico* dataset. Statistical significance was assessed using the Mann–Whitney *U*‐test. **p < 0.01. (D, E) Significant association between elevated KLK7 mRNA expression and shorter progression‐free survival in publicly available datasets. Affymetrix data were screened for KLK7 mRNA expression using probe ID 239381_at. Patients were categorized into stages 1 and 2 (D) and stages 3 and 4 (E), resulting in 600 and 472 cases, respectively. The cut‐off for the Kaplan–Meier estimation was set to 50% for stages 1 and 2, and 25% for stages 3 and 4. Overall survival between these groups was assessed using the Kaplan–Meier estimation and log‐rank test. *P < 0.05 for stage 1–2; *P* > 0.05 for stage 3–4.

To examine KLK7 protein expression in CRC, immunohistochemical (IHC) analysis was performed on paraffin‐embedded specimens, from a previous cohort of 38 CRC patients described elsewhere [14]. Representative images of the IHC staining are illustrated in Fig. 1A, demonstrating increased KLK7 expression in CRC tissues, localized in the cytoplasm of tumor cells. Additionally, the percentage of stronger KLK7 immunoreactivity correlated with advanced disease stages (Fig. 1B). These findings indicate that KLK7 expression is linked to the tumor‐advanced disease of CRC.

*In silico* analyses using the publicly available TCGA dataset confirmed significant differences in KLK7 expression between the CRC tissues of different grades. Specifically, there is a notable difference in KLK7 expression between stage 3 and 4 tumors compared to stage 1 and 2 tumors, with a *p*‐value of 0.002 (Fig. 1C). This suggests that KLK7 expression levels may correlate with tumor grade, potentially serving as a biomarker for tumor aggressiveness in CRC.

In the TCGA cohort, distinct associations were observed between KLK7 expression and progression‐free survival (PFS) in CRC patients. Higher KLK7 expression was significantly associated with reduced PFS in stage 1 + 2 tumors (*P* = 0.041), whereas in stage 3 + 4 tumors, only a trend towards significance was observed (*P* = 0.088) (Fig. 1D,E). This observation is not surprising, as higher stages of colorectal cancer already represent a highly aggressive disease state, driven by multiple factors, including comorbidities that can accelerate disease progression.

### KLK7 measurement in ascitic fluids from human peritoneal colorectal cancer metastasis

**Fig. 2:**
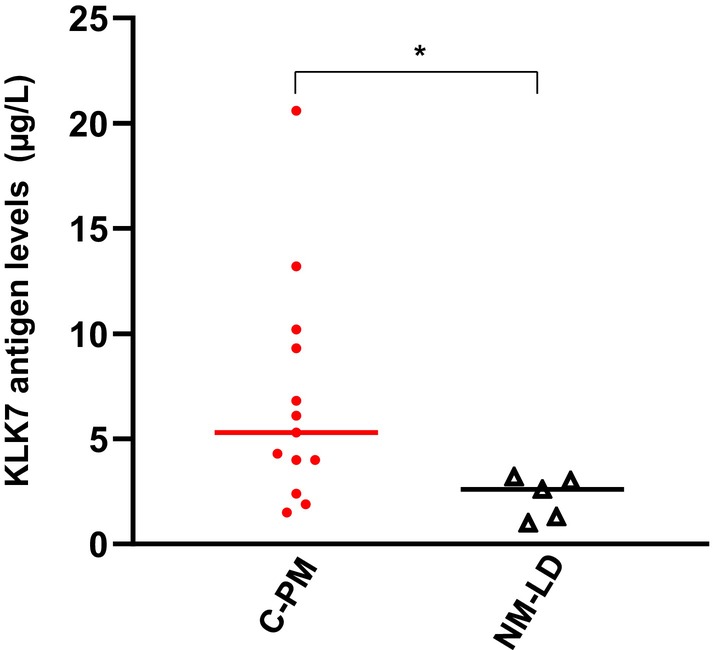
Immunodetection of human KLKs in ascitic fluids from patients with colorectal cancer peritoneal metastasis. Ascitic fluids were collected from patients with colorectal peritoneal metastasis (CPM) or from patients with nonmalignant liver diseases (NM‐LD). KLK7 antigen levels were evaluated by a sandwich‐type ELISA performed in duplicate for each sample. Data are presented as median (range) of KLK7 protein concentrations (μg·L^−1^). Statistical comparison between groups was performed using the Mann–Whitney *U*‐test. *P < 0.05.

Increased KLK7 expression is linked with poor prognosis of colorectal cancer [15, 30]. Therefore, we evaluated whether KLK7 may be involved in the peritoneal metastatic niche of CRC. This was done by measuring the levels of KLK7 by ELISA in 13 samples of ascitic fluids from a different cohort of patients with peritoneal colon cancer metastasis, and compared those with levels determined in patient samples with nonmalignant liver disease. As shown in Fig. 2, KLK7 levels appeared elevated in the ascitic fluid of patients with peritoneal colon cancer metastasis. Although one sample showed a markedly high concentration (> 20 μg·L^−1^), which may represent an outlier, the overall trend remained consistent and most values fell within a range of 2–10 μg·L^−1^. The median KLK7 concentration in this cohort was 5.3 μg·L^−1^, with a range of 1.5–20.6 μg·L^−1^ (Fig. 2). In contrast, patients with liver cirrhosis displayed much lower levels, with a median of 2.15 2.15 μg·L^−1^ and a range of 1.2–3.0 μg·L^−1^ (Fig. 2).

### Generation of a KLK7‐overexpressing colon cancer cell line

**Fig. 3:**
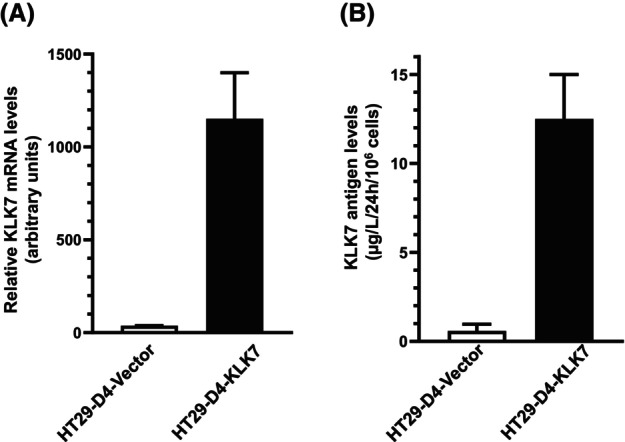
Quantification of *KLK7* mRNA levels *and measurement of KLK7 protein* secretion in HT29‐D4 colon cancer‐derived cell lines. HT29‐D4 cells were stably transfected with the KLK7 expression plasmid pRcRSV‐KLK7 (HT29‐D4‐KLK7) or with the pRcRSV vector alone (HT29‐D4‐Vector). HT29‐D4‐KLK7 represents a pool of KLK7‐transfected HT29‐D4‐derived cell line. (A) Total RNA from either HT29‐D4‐KLK7 or HT29‐D4‐Vector cells was reverse transcribed and subsequently analyzed by QPCR to quantify the *KLK7 mRNA* expression levels. GAPDH *mRNA* was used as the house keeping gene for normalization of the *KLK7* mean *mRNA expression*. Data are presented as mean ± SD from three independent experiments in duplicates. (B) Conditioned medium was collected from HT29‐D4‐KLK7 or HT29‐D4‐vector cells after 24 h of culture in a 12‐well plates and KLK7 antigen levels were assessed by ELISA. Data are presented as mean ± SD from three independent experiments in duplicates.

To analyze the biological function of KLK7 in CRC metastasis *in vitro* and *in vivo*, we generated expression vectors and stably overexpressed wild‐type KLK7 (HT29‐D4‐KLK7) and in some cases, as an additional control, an enzymatically inactive KLK7 mutant KLK7‐S/A (HT29‐D4‐KLK7‐S/A). As shown in Fig. 3, real‐time quantitative PCR (qPCR) analysis revealed that HT29‐D4‐KLK7 pool cells express high amounts of KLK7 mRNA, whereas HT29‐D4‐vector only cells express no or little KLK7 mRNA (Fig. 3A). We also measured the protein expression levels by ELISA and showed that HT29‐D4‐KLK7‐transfected cells exhibit KLK7 antigen levels (~ 13 μg·L^−1^) secreted in conditioned medium (Fig. 3B), which are in the same range as those found in many other colorectal cell lines [14]. In contrast, no or minimal immunoreactive KLK7 was detected in the conditioned medium of HT29‐D4‐vector control cells.

### KLK7 induces peritoneal metastasis of colon cancer cells in an *in vivo* model

**Fig. 4:**
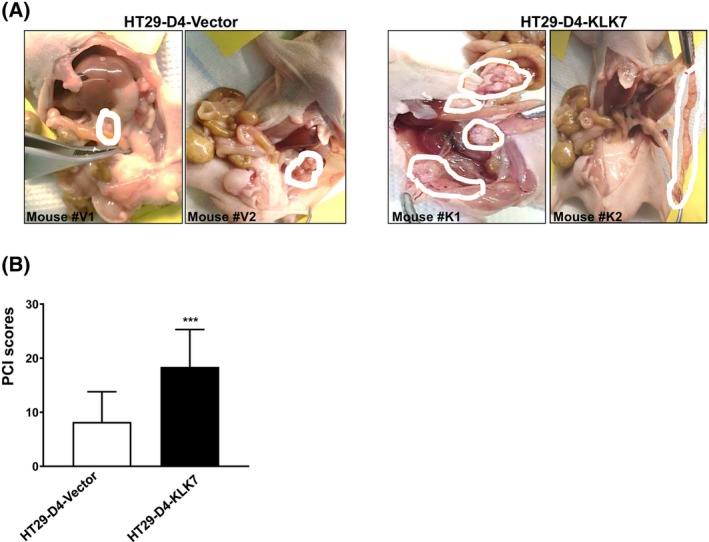
KLK7 overexpression mediates metastasis in an *in vivo* model for peritoneal metastasis. HT29‐D4‐KLK7 and HT29‐D4‐D4‐vector cells (1 × 10^6^ cells) were injected intraperitoneally (i.p) to BALB/c nude mice (*n* = 10 mice/group). Mice were sacrificed 42 days post‐injection to assess for peritoneal metastasis. (A) Abdominal cavity of the mice inoculated with HT29‐D4 tumor cells. White circles depict the locations of tumor nodules in various regions of the peritoneal wall, mesentery, and colon. (B) Measurement of tumor burden was quantified based on a modified peritoneal carcinomatosis index (PCI) score and presented as total PCI score. Total PCI is calculated by adding the scores of all regions and ranges from 0 to 27. Data represents mean ± SD (*n* = 10). Statistical comparison between groups was performed using the Mann–Whitney *U*‐test. ***P < 0.001.

To investigate the effect of KLK7 overexpression on colorectal cancer metastasis *in vivo*, we intraperitoneally injected HT29‐D4‐KLK7 cells and HT29‐D4‐vector cells into nude mice, utilizing this setup as a model for peritoneal metastasis. After 42 days, metastasis in the peritoneum was assessed macroscopically and quantified using the Peritoneal Cancer Index (PCI) score, a semi‐quantitative measure of intraperitoneal tumor load as described by [27]. As illustrated in Fig. 4, mice injected intraperitoneally (*i.p*.) with HT29‐D4‐KLK7 cells developed significantly more intra‐abdominal tumor nodules compared to those injected with control cells (Fig. 4A). Correspondingly, the PCI score, which indicates the extent of tumor growth in colorectal cancer, was significantly higher in the KLK7‐overexpressing group than in the control group (Fig. 4B).

These findings support the hypothesis that KLK7 overexpression in colon cancer cells enhances their dissemination and invasion, contributing to peritoneal metastasis.

### KLK 7 stimulates proliferation and colony formation of human colon cancer cells *in vitro*

**Fig. 5:**
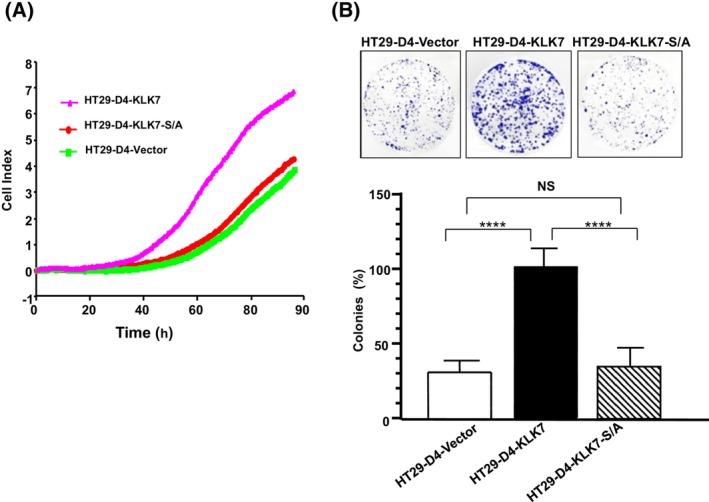
Overexpression of KLK7 increases colon cancer cell proliferation and clonogenicity. (A) Cell proliferation cells was monitored by using the XCELLigence real‐time analysis system. HT29‐D4 cells overexpressing wild‐type HT29‐D4‐KLK7, or its active site mutant (HT29‐D4‐KLK7‐S/A), or HT29‐D4‐vector cells were adjusted to 20 000 cells·mL^−1^ of complete medium and 100 μL of the cell suspensions were seeded into the wells of the E‐plate. The cell index (CI), based on impedance measurements was recorded every 15 min for up to 90 h. Data shown are from one representative experiment out of three independent replicates. (B) HT29‐D4‐KLK7, or its active site mutant (HT29‐D4‐KLK7‐S/A), or control cells (HT29‐D4‐vector cells) were plated at low density (1000 cells/well) in 6‐well plates and allowed to generate single colonies for 14 days. KLK7 induced a strong increase in colon cancer cell colony formation. Representative images were captured (upper panel) and quantified (lower panel) using an Azure™ Imaging Systems. Columns represent the mean percentage of colonies ± SD of three independent experiments. Statistical comparisons were performed using the Mann–Whitney *U*‐test. HT29‐D4‐KLK7 versus HT29‐D4‐vector cells, ****P < 0.0001; HT29‐D4‐KLK7‐S/A versus HT29‐D4‐vector cells, NS (*P* > 0.5).

To assess the impact of KLK7 activity on colon cancer cell behavior, we conducted several functional assays. We compared the growth of control HT29‐D4‐vector cells with both, wild‐type KLK7‐expressing HT29‐D4 cells and mutant KLK7‐S/A‐expressing HT29‐D4‐KLK7‐S/A cells. Cell proliferation was monitored in real time using impedance‐based cell index (CI) measurements every 15 min for up to 90 h. As shown in Fig. 5, the HT29‐D4‐KLK7 cells exhibited significantly faster growth (~ 2.5‐fold increase) compared to HT29‐D4‐vector cells. In contrast, the mutant HT29‐D4‐KLK7‐S/A cells did not display any significant increase in proliferation compared to the HT29‐D4‐KLK7 cells and the growth was comparable to HT29‐D4‐vector cells (Fig. 5A). These results suggest that the wild‐type KLK7 enzyme plays a key role in promoting colon cancer cell proliferation. Similarly, in an *in vitro* cell survival assay, which evaluates the ability of a single cell to grow into a colony, HT29‐D4‐KLK7 cells, but not HT29‐D4‐KLK7‐S/A cells, exhibited a significant increase in colony formation compared to HT29‐D4‐vector control cells (Fig. 5B). These findings suggest that KLK7 promotes cancer cell growth and colony formation through its enzymatic activity.

### KLK7 overexpression enhances HT29‐D4 cells migration

**Fig. 6:**
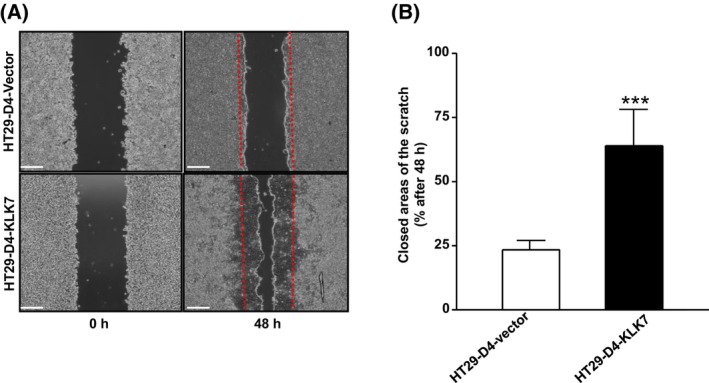
KLK7 overexpression mediates increased cell migration in HT29‐4 cell monolayers. Migration assays were conducted to assay the effect of KLK 7 on cell migration of CRC cells. (A) The wound space was photographed at 0 and 48 h. Scale bar = 250 μm. (B) Quantitative evaluation of the wound closure (%) expressed as the percentage of the wound area covered by the cells at 48 h after removal of the culture inserts (imagej software). Data are presented as the mean ± SD from six independent experiments. Statistical comparisons were performed using the Mann–Whitney *U*‐test. ***P < 0.001.

Since migration is a manifestation of tumor aggressiveness, we explored the effect of KLK7 on cell migration. We used a culture‐insert dish with two chambers separated by a wall. Once the cells reached approximately 90% confluence, the insert was removed, leaving a free gap. The initial gap of cells was recorded at 0 h, and then, the cells were cultured for an additional 48 h. As shown in Fig. 6, cell migration was significantly enhanced in HT29‐D4‐KLK7 cells compared to HT29‐D4‐vector cells (24 h: 5 ± 3% *vs* 30 ± 15%, *P* < 0.005; 48 h: 22 ± 2% *vs* 75 ± 10%, *P* < 0.0001). In HT29‐D4‐KLK7 cells, the coverage area was significantly larger compared to that of HT29‐D4‐vector cells (Fig. 6A). Within 48 h, HT29‐D4‐KLK7 cells have filled approximately 75 ± 10%, of the gap created by the insert, whereas control cells covered only 22 ± 2% (Fig. 6B). These findings indicate that KLK7 expression enhances migratory properties linked to tumor progression.

### KLK7 overexpression promotes spheroid formation *in vitro*

**Fig. 7:**
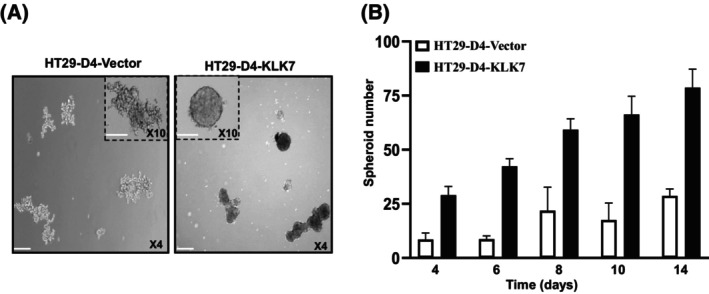
Effect of KLK7 overexpression in HT29‐D4‐derived colon cancer cells on spheroid formation. Spheroids were generated by growing cells at a clonal density of 1000 cells·mL^−1^ under neural crest cells culture conditions in low‐adherent plates as described in Material & Methods. Medium was changed twice a week. Spheres were allowed to grow over the course of 14 days. Spheres were counted and measured every 2–3 days under the Evos microscope. (A) Representative images of spheroids formed by HT29‐D4‐KLK7 and HT29‐D4‐vector cells randomly taken at day 10 after culturing. Scale Bar = 500 μm (4×). Inset (10×). Scale bar = 250 μm. (B) Number of spheroids formed by HT29‐D4‐KLK7 *versus* HT29‐D4‐vector cells. Data are shown as mean ± SD of at least three independent experiments each performed in triplicate.

Additional experiments were conducted to compare the spheroid formation capacity of HT‐29‐D4‐KLK7 cells versus HT‐29‐D4‐vector cells. As shown in Fig. 7, HT‐29‐D4‐KLK7 cells formed an increased number of larger and more compact spheroids, whereas HT‐29‐D4‐vector cells formed loosely organized spheroids (Fig. 7A). In fact, HT‐29‐D4‐KLK7 cells overexpressing cells are able to form spheres within 4 days under these culture conditions and the full spherical morphology is reached within 14 days. In contrast, HT‐29 cells transfected with the empty vector, (HT‐29‐D4‐vector) required a much longer time frame corresponding to 8 days (Fig. 7B). Moreover, HT‐29‐D4‐KLK7 cells showed a 3‐fold increase in spheroid numbers, reaching approximately 75 spheroids within 14 days (Fig. 7B). These data suggest that KLK7 induces colon cancer multicellular spheroids that may contribute to tumor cell survival during dissemination.

### KLK7 overexpression increases colon cancer cell adhesion

During our experiments, we observed that HT29‐D4‐KLK7 cells exhibited significantly greater attachment properties compared to control cells (HT29‐D4‐vector). To determine whether KLK7 overexpression in HT29‐D4 cells enhances colon cancer cell adhesion to ECM proteins, we conducted adhesion assays using selected basement membrane and ECM components, including type I, II, and IV collagens (Col‐I, Col‐II, Col‐IV), fibronectin (FN), laminin (LN), tenascin (TN), and vitronectin (VN); BSA was used as a negative control. As shown in Table 1, KLK7 overexpression significantly increased cell adhesion to nearly all ECM proteins, regardless of the absolute number of cells attached to each component (Table 1). These findings suggest that the enhanced proliferation of HT29‐D4‐KLK7 cells may be partially attributed to their increased adhesive capacity to specific ECM components.

**Table 1:** Effects of KLK7 overexpression on relative adhesion of HT‐29‐D4 cells to various ECM components.

ECM	HT29‐vector *n* = 6	HT29‐KLK7 *n* = 6	*P*
COL‐1	0.4343 ± 0.20	1.189 ± 0.1	< 0.0001
COL‐II	0.4647 ± 0.14	1.137 ± 0.22	0.0001
COL‐IV	0.3112 ± 0.080	0.812 ± 0.13	0.0091
FN	0.1372 ± 0.04	0.870 ± 0.13	0.0026
LN	0.7148 ± 0.080	1.265 ± 0.09	0.0002
TN	0.0270 ± 0.020	0.650 ± 0.16	0.0017
VN	0.0417 ± 0.010	0.407 ± 0.080	0.012

As described in M&M, cells were seeded in 96‐well plates coated with type I collagen (Col‐I), type II collagen (Col‐II), type IV collagen (Col‐IV), fibronectin (FN), laminin (LN), tenascin (TN), or BSA (B) as a negative control. After incubation for 2 h at 37 °C, non‐adherent cells were washed off and adherent cells were fixed, stained, solubilized, and quantified by measuring optical density at 570 nm. The results are expressed as mean ± SD of OD values normalized to BSA. Statistical differences were assessed using an unpaired *t*‐test with Welch's correction.

### KLK7 overexpression induces changes in cell morphology and moesin (MSN) expression

**Fig. 8:**
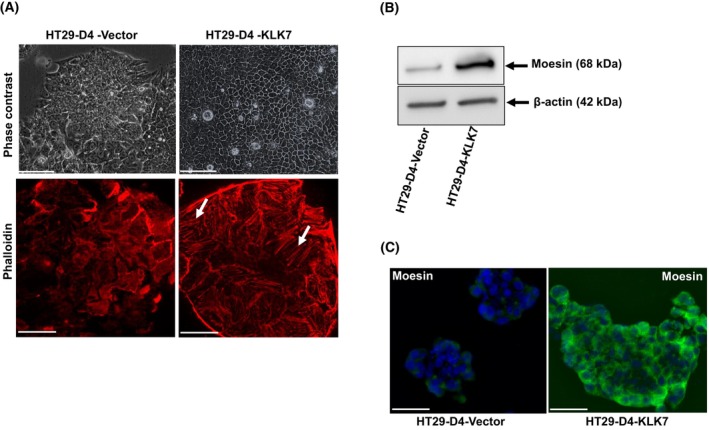
Effects of KLK7 overexpression on cell morphology, cytoskeletal rearrangements, and moesin expression. (A) HT29‐D4‐KLK7 *and* HT29‐D4‐vector cells were seeded onto coverslips and allowed to attach for 5–7 days, fixed and F‐actin was stained with phalloidin‐rhodamin. Visualization of the cell morphology was done by phase‐contrast microscopy of HT29‐D4‐KLK7 *and* HT29‐D4‐vector cells (upper panel) (Original magnification ×200). Representative fluorescence images of F‐actin filaments labeled with phalloidin‐rhodamin obtained by using maximum intensity projections by confocal microscopy (lower panel) (Original magnification ×630). Scale bar = 25 μm. Data are representatives at least three independent experiments. (B) Effect of KLK7 overexpression on moesin expression. Cell lysates from HT29‐D4‐KLK7 and HT29‐D4‐vector cells were analyzed by western blot for moesin expression using a rabbit anti‐moesin antibody. Membranes were probed with a mouse monoclonal anti‐beta‐actin antibody as loading control. Results are representative of three independent experiments. (C) Representative confocal photomicrographs of moesin in HT29‐D4‐KLK7 and HT29‐D4‐vector cells. Cells were fixed using 4% paraformaldehyde, permeabilized and immunostained with moesin polyclonal antibodies. (Original magnification ×630). Scale bar = 100 μm. Results are representative of two independent experiments.

Phase‐contrast imaging showed that HT29‐D4‐KLK7 overexpressing cells exhibited a distinct morphological change compared to control cells. While control cells appeared tightly packed, HT29‐D4‐KLK7 cells adopted a more organized, polygonal shape with refringent plasma membranes (Fig. 8A, upper panel). To confirm this observation, we performed F‐actin staining with rhodamin‐phalloidin and confocal microscopy demonstrated increased F‐actin organization into stress fibers in HT29‐D4‐KLK7 cells compared to HT29‐D4‐vector cells (Fig. 8A, lower panel). These observations suggest that KLK7 influences actin cytoskeleton reorganization and/or regulation of its binding proteins.

Previous studies have shown that moesin (MSN), a member of the ERM (ezrin‐radixin‐moesin) family, plays a crucial role in cancer progression and prognosis, with its expression levels correlating with patient outcomes across multiple cancer types. Given its involvement in cytoskeletal rearrangements and its role in regulating cell morphology, migration, and cancer progression [23], we examined MSN expression in HT29‐D4 cells overexpressing KLK7 using western blot analysis and immunofluorescence: KLK7 overexpression significantly increased MSN protein levels compared to HT29‐D4‐vector cells (Fig. 8B,C). These findings suggest that KLK7 influences MSN expression, thereby impacting cell morphology, actin cytoskeleton reorganization, cell migration, and cell adhesion.

### KLK7 effects on integrin expression pattern

**Fig. 9:**
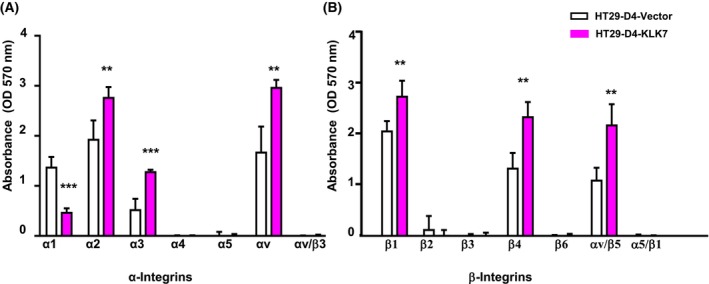
KLK7 overexpression in HT29‐D4 cells modifies cell surface expression of various integrin subunits. Integrin expression on the cell surface of HT29‐D4‐KLK7 and HT29‐D4‐vector cells was assessed by an integrin‐based cell adhesion array kit as described in Materials and Methods. KLK7 overexpression HT29‐D4 resulted in a significant decrease in the levels of α_1_ but increased the levels of α_2_, α_3_, α_v_, β_1_, β_4_, and α_v_β_5_‐integrins but not β_3_ integrin subunits. Data are presented as the mean ± SD from three independent experiments performed in duplicates. Statistical comparisons were performed using an unpaired *t*‐test with Welch's correction. **P < 0.01; ***P < 0.001.

We next aimed at investigating whether the increased adhesive capacity observed with KLK7 overexpression was associated with alterations in cell surface integrin expression, initially employing an integrin‐based cell adhesion array. As shown in Fig. 9, HT29‐D4‐KLK7 cells mediated significant alterations in integrin subunit levels compared to control HT29‐D4‐vector cells. Specifically, α1 integrin expression decreased, while α2, α3, αv, and β5 integrins increased (Fig. 9A). Additionally, β1, β4, and αv/β5 integrins were also elevated. β2 integrin levels remained unchanged, β3 integrin expression was weak in these cells, and α5 integrin was not expressed in the HT29‐D4 clone, consistent with previous reports (Fig. 9B) [31].

Interestingly, integrin β1, β4, and αv/β5 expression was increased in HT29‐D4‐KLK7 cells compared to HT29‐D4‐vector control cells. These findings suggest that the enhanced adhesive capacity of KLK7‐expressing HT29‐D4 cells could at least partially be due to increased levels of integrin β1, β4, αv, and integrin β5 in these cells.

**Fig. 10:**
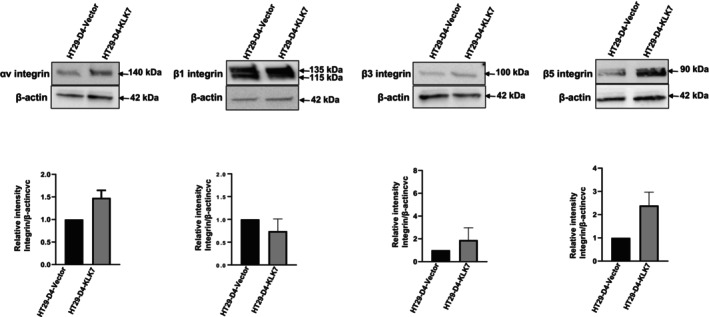
KLK7 overexpression in HT29‐D4 cells protein affects the expression of integrin subunits. Membrane‐rich lysates HT29‐D4‐KLK7 and HT29‐D4‐vector cells were analyzed by western blot analysis for integrin expression using rabbit antibodies against integrin β1, integrin α5, integrin αv, integrin β3, integrin α4 and integrin β5). Membranes were probed with mouse monoclonal anti‐beta‐actin antibody as loading control. The results shown are representative of at least two independent experiments. Data are presented as the mean ± SD from three independent experiments.

Finally, integrin expression was also determined by western blot analyses in membrane‐enriched cell lysates. As shown in Fig. 10, KLK7‐overexpression significantly increased the expression of integrin αv, and integrin β5 compared to HT29‐D4‐vector cells. Other integrins, such as integrin β4 and β1, did not show significant changes in KLK7‐overexpressing cells using this method. These results are in line with the observed increase in cell membrane expression of αv/β5 in HT29‐D4‐KLK7 cells, supporting the idea that KLK7 promotes cell‐matrix adhesion remodeling, thereby enhancing the migratory and invasive potential of tumor cells.

## Discussion

Kallikrein‐related peptidases (KLKs) have been increasingly recognized as critical mediators of tumor progression and poor prognosis in various cancers. This study specifically examined KLK7 to determine its functional impact on colon cancer cell behavior and its role in the metastatic progression of colorectal cancer. Our results show that KLK7 overexpression promotes several aggressive tumor characteristics, including enhanced colon cancer cell proliferation and migration. It also results in forming more compact and higher numbers of spheroids, and increases cell adhesion to extracellular matrix (ECM) components. These changes are accompanied by increased expression of cell adhesion molecules and noticeable alterations in cell morphology. Through a combination of microscopy and biochemical approaches, we observed that KLK7 overexpression led to elevated levels of MSN, a member of the ERM (ezrin, radixin, MSN) protein family, which is known for its role in cell morphology by linking the actin cytoskeleton to the plasma membrane. This may help explain the cytoskeletal and morphological changes observed in the study.

Clinically, we observed elevated KLK7 protein levels in the ascitic fluid of CRC patients with peritoneal metastasis compared to samples from patients with nonmalignant liver disease. This finding may help distinguish malignant from benign ascites and also suggests a potential role for KLK7 in disease progression. Supporting this, KLK7 overexpression in a xenograft mouse model led to a marked increase in peritoneal dissemination of cancer cells and overall tumor burden. Together, these findings indicate that KLK7 promotes an aggressive phenotype in colon cancer cells and may play a critical role in peritoneal metastasis, highlighting its potential as a therapeutic target. A key limitation of the analysis of the ascitic fluids of patients is the small sample size, which precludes definitive conclusions. Because ascites are relatively rare in colorectal cancer patients with peritoneal metastasis, recruitment of suitable cases is particularly challenging, further restricting validation of our findings. Therefore, our results should be considered exploratory and hypothesis‐generating, providing an initial basis for future multi‐center collaborative studies to comprehensively evaluate the diagnostic and prognostic potential of KLK7 in colorectal cancer PM.

Recent studies have focused on the evaluation of KLK proteases as potential prognostic biomarkers in gastrointestinal malignancies, including colorectal cancer (CRC) [32]. A large‐scale analysis of RNA sequencing data from The Cancer Genome Atlas (TCGA) identified KLK7 together with KLK6, KLK8, and KLK10 as promising diagnostic biomarker candidates for colon adenocarcinoma [12]. Moreover, KLK7 has emerged as a valuable biomarker across multiple malignancies, further supporting its potential relevance in CRC [33, 34].

In the present study, we found a notable association between higher KLK7 staining intensity and advanced stages of colorectal cancer, even in the limited number of patient samples that we previously used [14]. These findings align with previous studies that have linked elevated KLK7 expression to colon cancer progression [30, 32]. Moreover, a recent meta‐analysis by Peng et al. [13] further supports the prognostic relevance of KLK7, showing that increased expression of KLK6, KLK7, and KLK10 is significantly correlated with reduced overall survival (OS) and disease‐free survival (DFS) in CRC patients. Elevated expression of KLK7 has also been associated with poor prognosis and more aggressive tumor phenotypes in other malignancies, including ovarian [11], pancreatic cancers [18, 19], and melanoma [25]. Collectively, these data highlight KLK7 as a promising prognostic biomarker and potential therapeutic target in colorectal cancer and possibly in other cancer types.

Peritoneal metastasis, which originates from several gastrointestinal and gynecological cancers, including colorectal cancer, is frequently linked to poor prognosis and signifies advanced stages of the disease. Patients displaying peritoneal metastasis have a worse prognosis compared to metastasis of other organs such as the liver and lung. Peritoneal metastasis can occur through various routes, including direct seeding from the primary tumor and dissemination via peritoneal fluid (ascites) [2, 35]. A common clinical feature of peritoneal dissemination is the accumulation of tumor‐derived ascitic fluid within the peritoneal cavity. As KLK7 is a secreted protease, it was not surprising that we detected KLK7 in ascitic fluid samples from patients with peritoneal metastases of colon cancer. The observed levels displaying a median of 5.3 μg·L^−1^ (range: 1.5–20.6 μg·L^−1^) are higher or comparable to those previously reported in ovarian cancer ascites, which range from 1 to 10 μg·L^−1^ [36]. To our knowledge, increased KLK7 protein levels in ascites associated with colon cancer metastasis have not been previously reported. KLK7 secretion may result from malignant tumor cells shedding into the peritoneal cavity during colorectal peritoneal metastasis. Supporting this premise, KLK7 has been reported to be secreted by cells isolated from the ascitic fluid of ovarian cancer patients, where it contributes to tumor progression and peritoneal dissemination [37]. These findings suggest that KLK7 expression in tumor tissue may be reflected in the ascitic fluid, potentially contributing to its overall negative prognostic implications. Our measurement by ELISA cannot distinguish between catalytically active KLK7 or inactive KLK forms, for example, due to its association with serine proteinase inhibitors in the ascitic fluid. Nonetheless, several proteases including serine proteases of the blood coagulation pathway and the urokinase‐type plasminogen activator, have been detected in active form in ascitic fluids of cancer patients [38]. Therefore, it is tempting to speculate that KLK7 can sustain its activity in the ascitic fluid of CRC patients with PM.

The observed correlation between elevated KLK7 expression and advanced stages of disease, as demonstrated in this study and previous research [30], indicates a possible interaction between KLK7 and specific oncogenes that drive colorectal cancer progression [39]. A similar functional relationship has been established for KLK6, another member of the KLK family, in colorectal cancer. Tumors with high KLK6 expression display a unique mutation profile, including alterations in KRAS and MUC16, which are known to facilitate colorectal cancer progression [40]. Additionally, the gene regulatory roles of KLK4–7 have been documented in ovarian cancer, where they are associated with the upregulation of genes linked to cell invasion and metastasis, such as MSN and keratin 19 [41]. Overall, while the exact mechanisms and interactions of KLK7 with oncogenes in colorectal cancer need to be investigated, the existing evidence points to a significant role for KLK7 in cancer progression and metastasis.

Within the present study, we aimed at establishing a murine model of PM that closely mimics the metastatic spread observed in CRC patients. We used the cell line HT29‐D4, a well‐characterized stable epithelial subclone of HT29, reducing batch‐related heterogeneity. HT29‐D4 is known for its rather low aggressiveness, making it a suitable baseline model for studying the impact of increased KLK7 expression on tumor growth and invasiveness. Tumor nodules were detected in multiple organs, including the pancreas, intestines, stomach, omentum, and liver. To quantify metastatic spread, we applied a scoring system analogous to the Peritoneal Cancer Index (PCI) used in clinical studies [42]. We found a higher PCI and larger nodule sizes in mice injected with HT29‐D4‐KLK7 cells compared to those injected with HT29‐D4 cells containing the empty vector only. This observation aligns with findings from Loessner et al. [43] who reported that simultaneous overexpression of KLK4‐7 in ovarian cancer cells significantly increased tumor burden and metastatic spread in a tumor xenograft model. These results suggest that KLK7 may play a general role in enhancing tumor metastasis across various cancer types; thus, it is a key regulator of cancer cell migration and invasion indicating a potential general contribution of KLK7 to tumor metastasis across various cancer types.

Cancer peritoneal metastasis is a multistep process that begins with the detachment of cancer cells from the primary tumor and acquisition of motility. Our findings demonstrated that KLK7 overexpression in HT29‐D4 cells significantly enhanced colon cancer cell motility, as shown in different migration assays. These results are in line with previous studies showing that KLK7 enhances migration and invasion across various cancer types, including thyroid papillary carcinoma [44], gastric cancer [45], melanoma [25], and pancreatic cancer [18, 33]. In ovarian cancer, KLK7 is part of the cell secretome and is involved in cell migration processes, as determined by proteomics approaches [46]. After detaching from the primary tumor, cancer cells must escape cell death to survive within the peritoneal environment. Our findings indicate that KLK7 overexpression not only enhances cell proliferation but also improves survival, as evidenced in colony formation experiments.

A growing body of evidence suggests that tumor cells do not necessarily travel as single cells during the metastatic process, but often as clusters [47], that is, cells adhere to form spheroids, allowing them to evade anoikis and resist therapies [2, 48]. Our findings show that KLK7 overexpression enhances spheroid formation, consistent with its role in promoting multicellular aggregation and chemotherapy resistance in ovarian cancer [11]. Additionally, recent research shows that silencing KLK7 in triple‐negative breast cancer increases sensitivity to COX‐2 inhibitors [49], highlighting its role in drug resistance across cancer types.

KLK7 overexpression also enhances tumor cell adhesion to the extracellular matrix, facilitating implantation at metastatic sites. This is further supported by its ability to increase cell proliferation, as shown in the present study and also previous studies linking aberrant KLK7 expression to enhanced proliferation in colon cancer cells [14].

KLK7 plays a significant role in enhancing the aggressiveness and metastatic potential of colon cancer cells primarily through its proteolytic activity, which remodels the tumor microenvironment. This remodeling facilitates tumor progression by influencing gene regulation, modulating hormones, and growth factors under various environmental conditions, all of which are crucial for tumor growth, invasion, and metastasis [8]. Consequently, KLK7 may facilitate cancer cell dissemination directly or through its downstream substrates. KLK7 cleaves several substrates, such as fibronectin [50], tenascin‐C, CYR61, and midkine [51], which contribute to extracellular matrix remodeling and enhance tumor cell dissemination. Recent proteome analysis in ovarian cancer cell lines has identified pro‐MMP10, thrombospondin1, and IGFBP6 as putative KLK7 substrates [46], further underscoring its role in tumor microenvironment modification and metastasis.

KLK7 is embedded in proteolytic networks, that further modify the extracellular matrix composition. It has also been shown to cleave pro‐MMP9 to generate active MMP9 [52], a member of the MMP family, whose overexpression is related to poor prognosis in colon cancer patients [53]. These findings highlight KLK7's pivotal role in promoting metastasis through its extensive involvement in proteolytic processes and extracellular matrix remodeling.

While the majority of KLK7 functions involve proteolysis, KLK7 has been reported to exert non‐proteolytic functions in other contexts as well. For example, in ovarian cancer, a proteolytically inactive, N‐terminally truncated KLK7 form promoted tumor progression similarly to its active form, suggesting potential nonenzymatic roles in cancer [10, 11]. These findings indicate that while enzymatic activity is critical in our colon cancer model, non‐proteolytic mechanisms may contribute in other tumor types or biological contexts.

In contrast, our findings in the present study indicate that the pro‐tumorigenic effects of KLK7 in colon cancer cells are dependent on its proteolytic activity. Although direct enzymatic measurements were not feasible due to the lack of KLK7‐specific activity probes suitable for *in vitro* culture studies, overexpression of an active site mutant did not enhance cell growth or colony formation unlike the wild‐type enzyme. These results suggest that, in our experimental context, non‐proteolytic functions of KLK7 are unlikely to contribute significantly to the observed phenotypes.

Furthermore, while our overexpression studies in the mouse model demonstrate that KLK7 enhances tumor dissemination, we acknowledge that complementary *in vitro* invasion assays using selective KLK7 inhibitors could provide additional mechanistic insights. We have indeed identified potent and selective lead structures targeting KLK7, whereby their chemical properties are currently being optimized to enable effective inhibition in both *in vitro* and *in vivo* analyses. These future studies may help to further validate the role of KLK7 in cancer cell invasion and metastasis.

Confocal microscopy combined with rhodamine‐phalloidin staining revealed that KLK7‐overexpressing cells exhibited a more pronounced and organized arrangement of F‐actin stress fibers, indicating cytoskeletal remodeling associated with increased cell movement. Our results demonstrate that HT29‐D4‐KLK7 cells, compared to HT29‐D4‐vector cells, exhibit upregulated expression of MSN, a protein that cross‐links the plasma membrane, suggesting that KLK7 may facilitate colon cancer cell migration and invasion through MSN. Interestingly, combined overexpression of the human tissue kallikrein genes KLK4, 5, 6, and 7 has been shown to enhance the malignant phenotype of serous ovarian cancer cells [37] and, consistent with our findings, Wang et al. [41] identified MSN and KRT19 as downstream effectors of KLK4–7 in this cancer type. MSN has, in fact, been implicated in various cancers, interacting with multiple signaling pathways and regulating cell morphology [23], which supports its role in cancer cell migration and invasion, most likely by regulating the actin cytoskeleton [54]. Research has further demonstrated that MSN promotes CRC cell proliferation, adhesion, migration, and invasion, emphasizing its importance in cancer development and metastasis [23, 55]. Expression of MSN does not only support progression but is also associated with patient prognosis afflicted with several types of cancer, including CRC. Like KLK7, elevated MSN levels in CRC tissues are linked to more advanced tumor stages and poorer patient outcomes [56]. Interestingly, Lenos et al. [22] have provided evidence that MSN is necessary for peritoneal metastasis specifically in the molecular subtype CMS4 (Consensus Molecular Subtype 4). Whether KLK7 and colon cancer subtype CMS4 correlate remains to be investigated. This analysis could help determine whether shared pathways exist between CMS4‐related characteristics and KLK7 expression, potentially leading to new therapeutic targets or prognostic markers for aggressive colon cancer subtypes. The connection between KLK7 and MSN is particularly intriguing, as both proteins have been independently linked to increased cancer cell motility and invasiveness as demonstrated in this study and others [56].

Evidence suggests that KLK7‐induced MSN expression may be regulated by the Wnt/β‐catenin pathway. Studies have shown that MSN promotes CRC cell proliferation, adhesion, migration, and invasion through activation of the β‐catenin pathway [56]. Increased MSN expression in glioblastoma has been shown to activate the Wnt/β‐catenin pathway, promoting a more aggressive orthotopic tumor growth in nude mice [57]. Since KLK7 has been identified as an interacting partner of KLK6 and is highly co‐expressed in colon cancer [40], it is plausible that KLK7 similarly contributes to CRC progression potentially by modulating the Wnt/β‐catenin pathway through MSN regulation. While, in our study, the expression of the epithelial cell adhesion molecule (EpCAM) remained unchanged between KLK7‐overexpressing and control cells (Fig. S2), integrin array analysis revealed notable alterations in specific integrin subunits. In particular, expression of integrin α1 was reduced, whereas levels of α2, α3, αv, β1, β4, and β5 were elevated in KLK7‐overexpressing cells. Notably, KLK7‐overexpressing cells exhibited increased membrane expression of the αv/β5 heterodimer, a key mediator of cell adhesion. Western blot analysis further confirmed marked elevation of αv, β5, and β3 subunits, while the levels of β1 and β4 subunits remained unchanged—potentially reflecting differences in antibody specificity and detection sensitivity in both methods. In CRC, integrin αvβ5 is known to be expressed on tumor cells and contributes to the regulation of cell adhesion, migration, and invasion [35]. While direct evidence linking KLK7 to α5β1 integrin activation was reported in ovarian cancer [10], similar mechanisms likely apply to integrin subunits in colorectal cancer, given the overlapping roles of integrins in cell adhesion and signaling. However, integrin expression varies across cell types; therefore, KLK7 may engage different integrins in metastasis depending on the cancer type. KLK7 likely promotes expression and activation of certain integrin subunits in CRC by remodeling the ECM and enhancing the availability and function of integrin subunits, thereby supporting tumor cell adhesion, migration, and invasion. Direct mechanistic studies in CRC are still needed to fully delineate this pathway.

Collectively these findings suggest that KLK7 contributes to the key steps of colon cancer dissemination by upregulating MSN and modulating cell surface receptors. This functionality makes it a suitable agent both for monitoring disease progress as well as a potential target for therapy.

## Conclusion

Our study provides preliminary evidence that KLK7 may be a contributing factor in the metastasis of colorectal cancer (CRC), particularly in promoting peritoneal dissemination. Its presence in ascitic fluid and its association with aggressive cancer phenotypes suggest a potential, yet to be further validated, role as a biomarker for tracking disease progression. Disrupting KLK7 pathways may potentially mitigate the spread of CRC to the peritoneum, which remains a major clinical challenge. While preclinical evidence supports this mechanistic link, further clinical research with larger, multi‐center cohorts are needed to determine whether KLK7 could serve as a reliable biomarker for distinguishing malignant from benign ascites and for and for monitoring disease progression. Additional investigations into the diagnostic performance of KLK7 protein along with complementary functional analyses could strengthen the biological and clinical relevance of these findings and help evaluate the therapeutic potential of targeting KLK7 in CRC.

## Conflict of interest

The authors declare no conflict of interest.

## Author contributions

YZH performed *in vitro* experiments, image analysis and editing the manuscript. TD conducted *in silico* analyses using the TCGA dataset, performed control experiments, data review and editing the manuscript. MN performed western blot experiments. NAI discussed the data, performed data review and editing the manuscript. RLD provided clinical samples. VM discussed the data, performed data review and editing the manuscript. DD conceptualization, designed the study performed experiments interpreted data and wrote the manuscript. All authors contributed to the article and approved the submitted version.

## Supporting information


**Fig. S1.** Peritoneal Cancer Index (PCI) scoring system.
**Fig. S2.** Effect of KLK7 overexpression on EpCAM expression.

## Data Availability

The data that support the findings of this study are available from the corresponding author dalila.darmoul@inserm.fr upon reasonable request. This study uses publicly available datasets obtained from the KM Plotter platform (https://kmplot.com). The underlying expression and survival datasets hosted by KM Plotter originate from publicly accessible repositories including GEO, TCGA, and other microarray/RNA‐seq studies.
