# Supplementary material for: KLK7 overexpression promotes an aggressive phenotype and facilitates peritoneal dissemination in colorectal cancer cells
Source: FEBS Open Bio. 2025 Dec 3;16(5):1000–19. doi: 10.1002/2211-5463.70171 (PMC13145359; doi:10.1002/2211-5463.70171)
Supplement: Supplementary file 1 — Fig. S1. Peritoneal Cancer Index (PCI) scoring system. Fig. S2. Effect of KLK7 overexpression on EpCAM expression. [file FEB4-16-1000-s001.pdf]

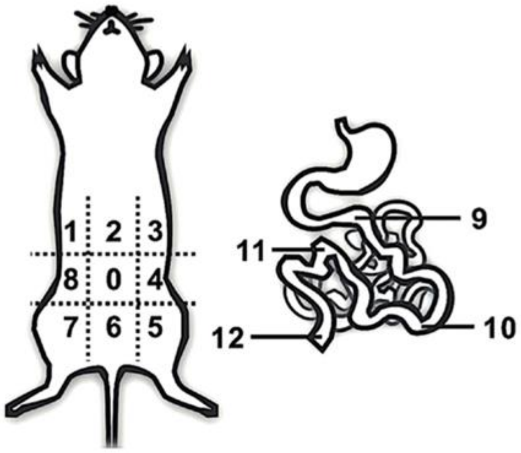

0	Central abdomen
1	Right upper quadrant
2	Epigastric region
3	Left upper quadrant
4	Left middle quadrant
5	Left lower quadrant
6	Pubic region
7	Right lower quadrant
8	Right middle quadrant
9	Proximal jejunum
10	Distal jejunum
11	Proximal ileum
12	Distal ileum

**Score**	**Tumor size**
0	no macroscopic lesion
1	Lesion from 1 to 2 mm, 1 to 2 sites
2	Lesion from 2 to 4 mm, 1 to 2 sites
3	lesion over 4 mm or more than 10 sites

Figure S1 : Peritoneal Cancer Index (PCI) scoring system

The PCI is a diagnostic and prognostic tool based on the sum of lesion scores across thirteen abdominal regions. Each region receives a score from 0 to 3 according to the size of the largest tumor deposit. The total PCI ranges from 0 to 27, with higher scores indicating more extensive and/or larger tumor involvement within the peritoneal cavity.

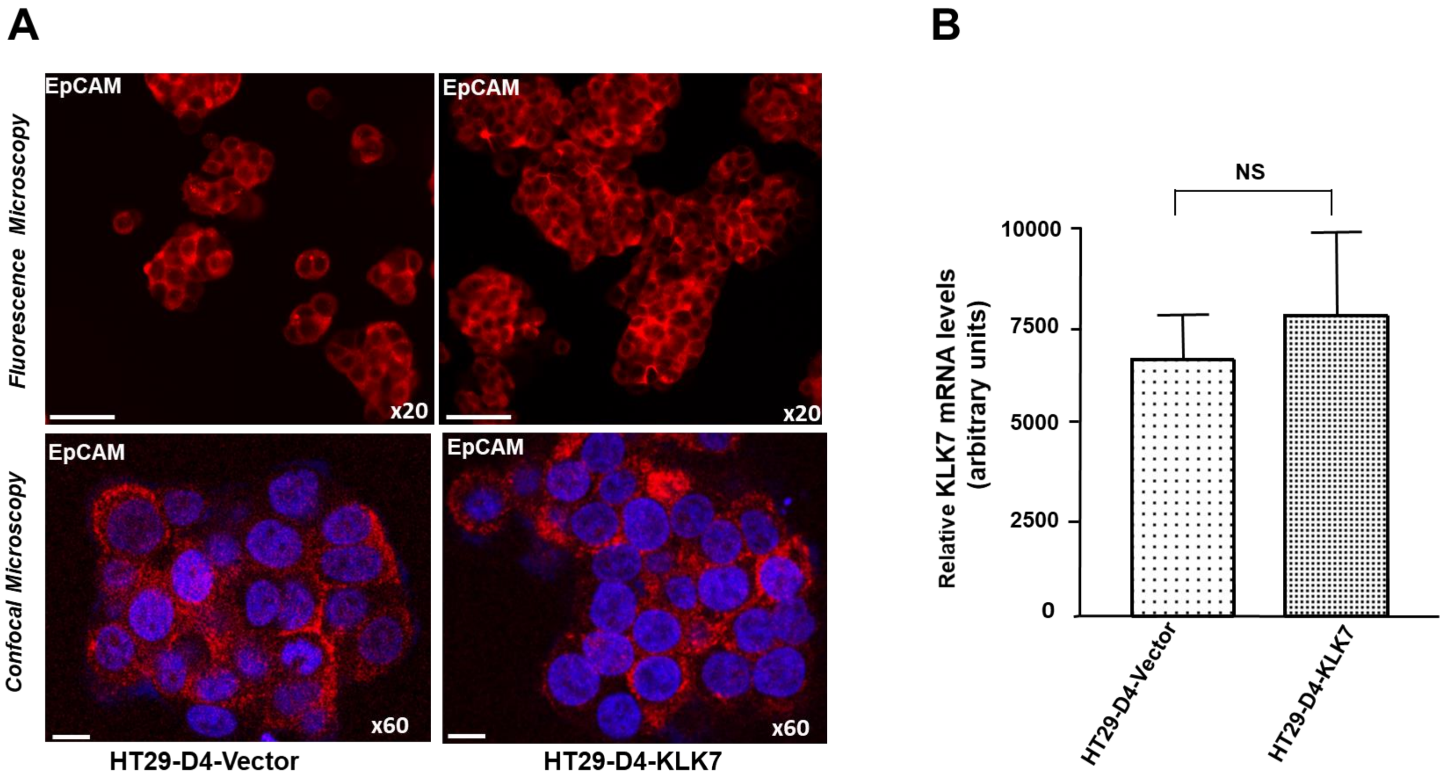

Figure S2 : Effect of KLK7 overexpression on EpCAM expression

A: HT29-D4-KLK7 and HT29-D4-vector cells were seeded onto coverslips and allowed to attach for 5-7 days. Cells were then fixed with 4% paraformaldehyde, permeabilized and immunostained for Epcam using a rabbit monoclonal antibody (#A1107 AB clonal, Cell Signaling). (Original magnification X 200).

B: Total RNA from either HT29-D4-KLK7 or HT29-D4-Vector cells was reverse transcribed and subsequently analyzed by QPCR to quantify the EpCAM mRNA expression levels. GAPDH mRNA was used as the house keeping gene for normalization of the. Data represents mean ± SD from three independent experiments performed in duplicates. Mann-Whitney U test was employed to compare EpCAM mRNA expression levels between HT29-D4-Vector cells *vs* HT29-D4-KLK7. NS > 0.5.
